# Quantitative copy number analysis by Multiplex Ligation-dependent Probe Amplification (MLPA) of BRCA1-associated breast cancer regions identifies BRCAness

**DOI:** 10.1186/bcr3049

**Published:** 2011-10-27

**Authors:** Esther H Lips, Nadja Laddach, Suvi P Savola, Marieke A Vollebergh, Anne MM Oonk, Alex LT Imholz, Lodewyk FA Wessels, Jelle Wesseling, Petra M Nederlof, Sjoerd Rodenhuis

**Affiliations:** 1 Department of Experimental Therapy, The Netherlands Cancer Institute-Antoni van Leeuwenhoek Hospital, Plesmanlaan 121, 1066 CX, Amsterdam, The Netherlands; 2 Department of Pathology, The Netherlands Cancer Institute-Antoni van Leeuwenhoek Hospital, Plesmanlaan 121, 1066 CX, Amsterdam, The Netherlands; 3 Department of Molecular Biology, The Netherlands Cancer Institute-Antoni van Leeuwenhoek Hospital, Plesmanlaan 121, 1066 CX, Amsterdam, The Netherlands; 4 Department of Bioinformatics and Statistics, The Netherlands Cancer Institute-Antoni van Leeuwenhoek Hospital, Plesmanlaan 121, 1066 CX, Amsterdam, The Netherlands; 5 Department of Medical Oncology, The Netherlands Cancer Institute-Antoni van Leeuwenhoek Hospital, Plesmanlaan 121, 1066 CX, Amsterdam, The Netherlands; 6 MRC-Holland, Willem Schoutenstraat 6, 1057 DN, Amsterdam, The Netherlands; 7 Department of Medical Oncology, Deventer Hospital, N. Bolkesteinlaan 75, 6416 SE, Deventer, The Netherlands

## Abstract

**Introduction:**

Our group has previously employed array Comparative Genomic Hybridization (aCGH) to assess the genomic patterns of BRCA1-mutated breast cancers. We have shown that the so-called BRCA1-like^aCGH ^profile is also present in about half of all triple-negative sporadic breast cancers and is predictive for benefit from intensified alkylating chemotherapy. As aCGH is a rather complex method, we translated the BRCA1^aCGH ^profile to a Multiplex Ligation-dependent Probe Amplification (MLPA) assay, to identify both BRCA1-mutated breast cancers and sporadic cases with a BRCA1-like^aCGH ^profile.

**Methods:**

The most important genomic regions of the original aCGH based classifier (3q22-27, 5q12-14, 6p23-22, 12p13, 12q21-23, 13q31-34) were mapped to a set of 34 MLPA probes. The training set consisted of 39 BRCA1-like^aCGH ^breast cancers and 45 non-BRCA1-like^aCGH ^breast cancers, which had previously been analyzed by aCGH. The BRCA1-like^aCGH ^group consisted of germline BRCA1-mutated cases and sporadic tumours with low BRCA1 gene expression and/or BRCA1 promoter methylation. We trained a shrunken centroids classifier on the training set and validation was performed on an independent test set of 40 BRCA1-like^aCGH ^breast cancers and 32 non-BRCA1-like^aCGH ^breast cancer tumours. In addition, we validated the set prospectively on 69 new triple-negative tumours.

**Results:**

BRCAness in the training set of 84 tumours could accurately be predicted by prediction analysis of microarrays (PAM) (accuracy 94%). Application of this classifier on the independent validation set correctly predicted BRCA-like status of 62 out of 72 breast tumours (86%). Sensitivity and specificity were 85% and 87%, respectively. When the MLPA-test was subsequently applied to 46 breast tumour samples from a randomized clinical trial, the same survival benefit for BRCA1-like tumours associated with intensified alkylating chemotherapy was shown as was previously reported using the aCGH assay.

**Conclusions:**

Since the MLPA assay can identify BRCA1-deficient breast cancer patients, this method could be applied both for clinical genetic testing and as a predictor of treatment benefit. BRCA1-like tumours are highly sensitive to chemotherapy with DNA damaging agents, and most likely to poly ADP ribose polymerase (PARP)-inhibitors. The MLPA assay is rapid and robust, can easily be multiplexed, and works well with DNA derived from paraffin-embedded tissues.

## Introduction

Breast cancers are genomically highly instable, and array Comparative Genomic Hybridization (aCGH) analysis shows numerous gains and losses of whole chromosomes or parts of chromosomes. Previous studies have shown that hereditary breast cancer tends to develop specific genomic alterations, based on which they can be distinguished from sporadic tumours [1-5]. Our group has employed aCGH to assess the genomic patterns of BRCA1-mutated breast cancers [6]. This has resulted in a BRCA1-like classifier based on specific aberrations of BRCA1-mutated breast cancers compared to sporadic tumours. This classifier allows identification of familial breast cancer cases in patients whose germ line BRCA1-mutation status is unknown [6] or assists in the classification of BRCA1 variants of unknown significance [6,7]. It is currently employed in routine diagnostics in our clinical genetic centre. The BRCA1-like aCGH pattern may also be present in sporadic breast cancers, which frequently show evidence of impaired BRCA1 function due to other causes than mutation, for example, methylation [8-10]. In fact, we and others have shown that sporadic basal-like breast cancers resemble BRCA1 mutated cancers in many different ways [11-13]. This concept of "BRCAness" in sporadic cancers is under study in many centres as it may have implications for treatment selection [14-16].

BRCA1 mutated cancers are more sensitive to DNA damage inducing chemotherapy than their sporadic counterparts [17]. This can be explained by their defective homologous recombination pathway, a DNA repair mechanism in which BRCA-proteins have a major role. In BRCA1-associated cancers, the homologous recombination defect renders tumours highly sensitive to chemotherapy that causes double strand breaks (DSBs) in the DNA replication phase of the cell cycle, when homologous recombination is the dominant DSB repair mechanism. This observation also explains the sensitivity of tumours in BRCA1 mutation carriers to the novel class of poly ADP ribose polymerase (PARP) inhibitors [15].

We have shown in two independent studies that approximately half of all triple-negative (TN) tumours have a BRCA1-like aCGH profile [9,10]. We have hypothesized that these tumours harbour a homologous recombination deficiency and thus would be very sensitive to DSB-inducing chemotherapy, similar to tumours arising in BRCA1-mutation carriers. In agreement with this assumption, we have demonstrated in a retrospective analysis of a randomized controlled trial that the so-called BRCA1-like^aCGH ^profile was strongly predictive for benefit from intensive carboplatin-based alkylating chemotherapy [10].

As the aCGH assay is a rather complex method, requiring a reasonable amount of high quality DNA and specialized equipment, we endeavoured to devise an alternative method to determine the BRCA1-like profile in breast cancers suitable for routine use in pathology laboratories. Moreover, a commercially available test would make it possible to implement this approach uniformly in pathology laboratories across various institutes and countries. To achieve this, we translated the BRCA1^aCGH ^profile to a Multiplex Ligation-dependent Probe Amplification (MLPA) assay. MLPA is a method based on amplification and relative quantification of the ligated adjacent probes, which can target up to 50 different genomic regions that show diagnostically or clinically significant copy number changes in patient samples [18]. It is a simple and robust assay, which can be highly multiplexed and requires only a small amount of input DNA that can easily be obtained from paraffin-embedded tumour material. These characteristics make MLPA an ideal method for a clinical application.

In this study, we test the performance of a MLPA-kit, by a direct comparison with aCGH BRCA1 classifiers scores. In addition, we show how this kit can be used to identify both BRCA1-mutated breast cancers and sporadic cases with a BRCA1-like^aCGH ^profile and we present evidence that the kit can be used for treatment selection.

## Materials and methods

### Patient selection

Four series of breast cancer specimens were employed: (i) 52 tumours from the NKI-clinical genetic centre, from which the BRCA1 mutational status was known. These patient samples had also been used to build the original aCGH BRCA1-like classifier [6]; (ii) 58 tumour samples from a neoadjuvant chemotherapy trial at the NKI-AVL [9]; (iii) 46 triple-negative tumour samples from a randomized study in The Netherlands that compared intensified alkylating chemotherapy with conventional dose chemotherapy [19]; and (iv) 69 triple-negative tumours, collected at the Deventer Hospital. All either participated in clinical studies, which had been approved by the institutional review board, or received chemotherapy according to the standard arm of one of these studies off protocol. The local medical ethics committee approved the study protocols. At the time of building the classifier, DNA from 84 samples from the first two sets was available, so these samples were used as the training set. To test the classifier in new samples, we collected 26 additional cases from the first two series and used all 46 samples from the randomized controlled trial. In addition, when the classifier was ready for prospective validation, we applied the MLPA kit on the independent set of 69 triple-negative samples. All tumours had previously been studied by aCGH, and thus BRCA1-like profile scores were available. See Table 1 for an overview of all samples used.

**Table 1:** Sample characteristics

	Training set		Test set		Extra Validation set	
	**n (%)**		**n (%)**		**n (%)**	
**Total**	84	100%	72	100%	69	100%
**Patient series**						
Clinical genetics centre	34	40%	18	25%		
Neoadjuvant series	50	60%	8	11%		
Randomised controlled trial series	0	0%	46	64%		
**Subtype**						
HER2+	11	13%	5	7%	0	0%
ER+HER2-	22	26%	10	14%	0	0%
Triple-negative (TN)	52	61%	57	79%	69	100%
**ER expression**						
Negative	57	67%	59	82%	69	100%
Positive	28	33%	13	18%	0	0%
**BRCA1 mutation**						
BRCA1 mutation	16	19%	17	24%	7	10%
No mutation	19	23%	44	61%	0	0%
Unknown	49	58%	11	15%	62	90%
**BRCA1like status aCGH**						
BRCA1-like	37	44%	40	56%	50	72%
Sporadic-like	47	56%	32	44%	19	28%

aCGH, array Comparative Genomic Hybridisation.

### DNA isolation

DNA from all tumours was available from previous studies [6,9,10]. Briefly, DNA from the clinical genetics series, the randomized trial series, and the Deventer series was isolated from formalin-fixed, paraffin-embedded (FFPE)-tumour tissue, using NaSCN and purified with a DNeasy column (Qiagen DNA extraction kit, Qiagen, Venlo, The Netherlands). DNA from the neoadjuvant chemotherapy study samples was isolated from fresh-frozen tumour tissue, using RNAzol (Tel-test) and Back Extraction Buffer (consisting of guanidine thiocyanate, sodium citrate, and Tris). As reference DNA, we used commercially available human Promega female DNA (Promega, Madison, WI, USA). DNA samples from all three breast cancer groups had previously been typed with a 3.5 k BAC array and results have been published before [6,9,10]. We used the original BRCA1-like classifier developed by Simon Joosse [6,9,10], which was adapted by Vollebergh *et al*. [10].

### MLPA

Multiplex Ligation-Dependent Probe Amplification (MLPA) is a high throughput, PCR based method to determine the relative copy number of various DNA target sequences in a small quantity of human DNA. It is based on the annealing of a mixture of oligonucleotides on their cognate DNA sequences. Each MLPA probe consists of two parts, and the longer part of the probe contains a stuffer DNA sequence of variable length (19 to 370 bp), which allows multiplex detection of amplification products (120 nt to 500 nt) using a capillary sequencer. The amount of amplification product is proportional to the number of target sequences and thus the number of target recognition sites can be quantified. The standard MLPA-protocol, which was used here, has been described elsewhere [18]. In short, 100 ng target DNA/5 μl of 10 mM Tris (pH 8), 0.1 mM ethylenediaminetetraacetic acid was denaturated for five minutes at 98°C after which 3 μl of the probemix mixture was added. The sample DNA and P376-B1 MLPA probemix mixture was heated at 95°C for 1 minute and incubated at 60°C overnight (16 h). Ligation was performed with the temperature-stable Ligase-65 enzyme (MRC-Holland, Amsterdam, The Netherlands) for 15 minutes at 54°C. Then the ligase was inactivated by incubation for five minutes at 98°C. Ten microliters of this ligation mix were premixed with 30 μl PCR buffer and transferred into a PCR machine at 60°C. Subsequently, a 10 μl mix was added containing deoxynucleoside triphosphate, Taq polymerase and one unlabeled and one carboxyfluorescein-labeled (FAM) PCR primer, which are complementary to the universal primer sequences present in all MLPA probes. PCR was carried out for 35 cycles (30 sec at 95°C, 30 sec at 60°C and 60 sec at 72°C). The fragments were analyzed on an ABI model 310 or 3700 capillary sequencer (Applied Biosystems, Nieuwerkerk aan den Ijssel, The Netherlands) using Genescan-ROX 500 size standards. Fragment analysis was performed using Genescan and Genotyper software (Applied Biosystems).

### MLPA data normalization

To automate the interpretation of the fragment analysis, the relative quantity of the amplified probes in each sample was determined using a template in Excel (Microsoft, Redmond, WA, USA) [20]. For this purpose, the relative peak areas for each probe were calculated as fractions of the total sum of peak areas in each sample. Subsequently, the fraction of each peak was divided by the average peak fractions of the corresponding probe in control samples (Promega female reference DNA).

### Class prediction

Data values from Excel for all the 34 target specific probes were used for prediction analysis for microarrays (PAM). In brief, the nearest shrunken centroids method [21] was applied to the training set of 84 tumours with maximum distinct clinical courses (first set) and the classification performance was evaluated by a 10-times-repeated 10-fold cross validation as described previously. Subsequently, the obtained classifier was tested on the 72-sample test set. The BAC aCGH BRCA1-classifier [6] (adapted by Vollebergh *et al*. [10]) was considered as the gold standard. For the MLPA classifier the cut-off value to classify a sample as 'BRCA1-like' was set at 0.5. Below this score, a sample was classified as 'Sporadic-like'.

## Results

### Translation of the aCGH BRCA1-like classifier to a MLPA kit

Different classifiers were developed over time, one consisting of 371 probes [6], and one containing 191 probes [6]. The top centroids were selected from the 191 classifier and genomic location of the BACs was listed (most centroids were in chromosome 3q22-27 (gain), 5q12-14 (loss), 6p23-22 (gain), 12p13 (gain), 12q21-23 (loss), and 13q31-34 (gain)) [6]. In addition, we added probes for 3p21 (loss), 10p13-15 (gain), 10q23 (loss) (PTEN region), 14q22-24 (loss), 15q15-21 (loss), as these regions also made an important contribution to the BAC-classifier. In those regions we searched for existing MLPA probes from the MRC-Holland MLPA probe database. We attempted to cover each genomic region with at least two, preferably three, MLPA probes, as the BAC clones were also quite large, and the BAC classifier covered, in general, large genomic regions. When no probes were available for a specific region, new probes were designed. This process resulted in several versions of the MLPA BRCA1-like kit. The kits were tested to evaluate the performance of the probes, also in DNA from normal tissue and in DNA from FFPE-derived tissues. The final version of the kit (fourth) gave the best performance and was used for further validation. Previous versions either did not cover every region by a minimum of two probes, contained probes not working in FFPE derived DNA, or contained probes which failed for another reason. The final version of the kit contained 34 target specific probes, corresponding to the BAC classifier regions, 7 reference probes, randomly spread over the genome and in regions not affected in breast cancer, 2 probes for BRCA1 and 2 probes for BRCA2, resulting in a total of 45 probes (Table 2). The correlation between a BAC probe and its corresponding MLPA probe ranged between 0.79 and 0.96. The average correlation between two or three MLPA probes spanning the same genomic area was 0.94.

**Table 2:** All probes of the BRCA1-like MLPA kit

Probe name	Genomic location	Stuffer length	Probe type
SEMA3B - 3	03-050282676	140	Test_loss
RASSF1 - 1	03-050353347	328	Test_loss
DEPDC1B - 2	05-060018734	241	Test_loss
MCCC2 - 16	05-070984253	409	Test_loss
GPR98 - 90	05-090495354	184	Test_loss
APC - 1	05-112071337	226	Test_loss
LMNB1 - 7	05-126184598	214	Test_loss
PTEN - 1	10-089614110	233	Test_loss
PTEN - 9	10-089714978	463	Test_loss
CEP290 - 26	12-087019051	400	Test_loss
BTG1 AREA - 29	12-090905485	155	Test_loss
PAH - 3	12-101812669	202	Test_loss
BMP4 - 4	14-053488631	454	Test_loss
GCH1 - 6b	14-054380537	283	Test_loss
RDH12 - 7	14-067263397	319	Test_loss
THBS1 - 1	15-037660496	346	Test_loss
CAPN3 - 16	15-040479002	148	Test_loss
FBN1 - 4	15-046679657	172	Test_loss
CASR - 7	03-123485226	483	Test_gain
ATR - 47	03-143651063	373	Test_gain
HLTF - 1	03-150286916	193	Test_gain
PDCD10 - 5	03-168905298	382	Test_gain
PIK3CA - 2	03-180399607	427	Test_gain
TPMT - 5	06-018247829	265	Test_gain
HFE - 2	06-026198995	301	Test_gain
FANCE - 7	06-035535433	274	Test_gain
GATA3 - 1	10-008136773	136	Test_gain
CUGBP2 - 6	10-011017023	364	Test_gain
FGF23 - 1	12-004358933	436	Test_gain
ETV6 - 5	12-011913651	474	Test_gain
ABCC4 - 23	13-094525703	247	Test_gain
PCCA - 6	13-099607548	418	Test_gain
PCCA - 11	13-099718959	291	Test_gain
FGF14 - 2	13-101366785	492	Test_gain
BRCA1 - 20	17-038462662	390	BRCA probe
BRCA1 - 2	17-038529544	167	BRCA probe
BRCA2 - 5	13-031798006	337	BRCA probe
BRCA2 - 11	13-031812003	130	BRCA probe
REFERENCE	02-071430713	355	Reference
REFERENCE	02-267097	310	Reference
REFERENCE	07-075448405	124	Reference
REFERENCE	07-116126400	160	Reference
REFERENCE	10-042932039	178	Reference
REFERENCE	10-075547782	220	Reference
REFERENCE	14-019994693	208	Reference

MLPA, Multiplex Ligation-dependent Probe Amplification

### BRCA1-like MLPA predictor

To build the BRCA1-like MLPA classifier we used a set of samples from BRCA1 mutation carriers and a set of sporadic tumours classified as BRCA1-like by the aCGH classifier. These 'positive' samples were compared with breast tumours that were known to have a sporadic-like profile by aCGH. Using this approach, we trained the classifier to identify tumours in mutation carriers and sporadic cases with a BRCA1-like profile. The aCGH BRCA1-score was considered as the gold standard and it was used for class labelling. The resulting training set consisted of 84 breast tumours: 39 BRCA1-like^aCGH ^and 45 Sporadic-like^aCGH ^tumours. We used Shrunken Centroids from the package PAM to discriminate between BRCA1-like and Sporadic-like tumours. A 10-fold cross-validation resulted in an optimal delta of 0, and this parameter setting resulted in the smallest number of misclassifications. As a consequence, all 34 test probes were used for further classification. The overall error rate in the training set was 6% (Table 3). See Table 4 for the top 10 centroids contributing to the MLPA classifier.

**Table 3:** Results from the 10-fold cross-validation on the training set

	*Predicted with MLPA*		
	BRCA1-like	Sporadic-like	Total
*aCGH result*			
BRCA1-like	36	3	39
Sporadic-like	2	43	45
Total	38	46	84
	Number	Percentage	
Error	5/84	6%	
Accuracy	79/84	94%	

aCGH, array Comparative Genomic Hybridisation; MLPA, Multiplex Ligation-dependent Probe Amplification

**Table 4:** Top centroids from the BRCA1 MLPA classifier

Rank	Probe name	Genomic position	BRCA1-like score	Sporadic score
1	MCCC2 - 16	05-070984253	-0.4351	0.3771
2	PAH - 3	12-101812669	-0.2951	0.2558
3	CAPN3 - 16	15-040479002	-0.2929	0.2538
4	BMP4 - 4	14-053488631	-0.2814	0.2439
5	PIK3CA - 2	03-180399607	0.2768	-0.2399
6	GCH1 - 6b	14-054380537	-0.2763	0.2394
7	DEPDC1B - 2	05-060018734	-0.265	0.2297
8	GATA3 - 1	10-008136773	0.2488	-0.2156
9	FBN1 - 4	15-046679657	-0.2485	0.2153
10	GPR98 - 90	05-090495354	-0.2458	0.213

MLPA, Multiplex Ligation-dependent Probe Amplification.

### Results test set prediction

The classifier trained on the training set was tested in a new group of 72 tumours. This test set consisted of (i) 18 tumours from the clinical genetics centre; (ii) 8 tumours from the neoadjuvant series; and (iii) 46 triple-negative tumour samples from a randomized controlled clinical trial that studied the efficacy of intensified alkylating chemotherapy in breast cancer. Inclusion of this last series in the test set enabled us to assess whether the MLPA classifier had the same value in chemotherapy benefit selection as the original aCGH assay (10). Of the 72 test set tumours, 62 samples were correctly classified and 10 were misclassified when the aCGH test was regarded as the gold standard, resulting in an overall error rate of 14% (Table 5). The sensitivity was 85% and the specificity was 87%.

**Table 5:** Classification results of the test set

	*Predicted with MLPA*		
	**BRCA1-like**	**Sporadic-like**	**Total**
*aCGH result*			
BRCA1-like	35	6	41
Sporadic-like	4	27	31
Total	39	33	72
	Number	Percentage	
Sensitivity	35/41	85%	
Specificity	27/31	87%	
PPV	35/39	90%	
NPV	27/33	82%	
Error	10/72	14%	
Accuracy	62/72	86%	

aCGH, array Comparative Genomic Hybridisation; MLPA, Multiplex Ligation-dependent Probe Amplification; NPV, negative predictive value; PPV, positive predictive value

### Prospective validation

We wanted to validate the classifier in an independent set of triple-negative samples, which can be viewed as a prospective validation. For this purpose, we used 69 triple-negative tumours from the Deventer Hospital, which had recently been typed by aCGH (manuscript in preparation). A total of 64 out of 69 tumours were correctly classified, resulting in an accuracy of 93% and an error rate of 7%. Three BRCA1-like^aCGH ^tumours were classified as Sporadic-like by MLPA and two Sporadic-like^aCGH ^tumours were typed as BRCA1-like.

### Performance of the classifier to detect BRCA1-mutation carriers and BRCA1 promoter methylation

By combining training, test set and the prospective validation set, we had 40 tumours from patients known to be BRCA1 mutation carriers. Five of these 40 tumours did not show a BRCA1-like pattern on MLPA and, similarly, five of the 40 were not BRCA1-like according to aCGH (Table 6). Two of the discordant tumours tested Sporadic-like in both the MLPA and the aCGH assay, whereas six of the BRCA1-mutation carriers were either classified Sporadic-like by MLPA or aCGH. Interestingly, one tumour which was classified as Sporadic-like by both MLPA and aCGH was hormone receptor positive, whereas all other BRCA1-mutated tumours were triple-negative.

**Table 6:** BRCA1-like profile is samples with a BRCA1- mutation, -methylation or low gene expression

BRCA1 classification	n (%)
**BRCA1 mutated by sequencing**	40
BRCA1-like by MLPA	35 (88%)
BRCA1-like by aCGH	35 (88%)
**BRCA1 promoter methylation**	20
BRCA1-like by MLPA	18 (90%)
BRCA1-like by aCGH	19 (95%)
**BRCA1 low gene expression**	13
BRCA1-like by MLPA	13 (100%)
BRCA1-like by aCGH	12 (92%)

aCGH, array Comparative Genomic Hybridisation; MLPA, Multiplex Ligation-dependent Probe Amplification

For a subset of samples we had methylation and real-time PCR data for BRCA1. We assumed that tumours with a BRCA1-like profile could have BRCA1 pathway inactivation due to other causes than a mutation. Twenty tumours showed BRCA1 promoter methylation. A total of 18 (90%) of these were BRCA1-like by MLPA, and 19 (95%) by aCGH (Table 6). Thirteen tumours showed low BRCA1 gene expression measured by qRT-PCR. These tumours all had a BRCA1-like pattern by MLPA, and all but one by aCGH.

### Ability of the MLPA-set to predict chemotherapy benefit

In a previous publication, we reported a significantly better survival in patients with a BRCA1-like^aCGH ^tumours treated with an intensified alkylating regimen compared to conventional dose chemotherapy [10]. To study the performance of the MLPA assay, all TN tumours from this randomized controlled trial from which a sufficient amount of DNA was available were included in the test set, resulting in a total of 46 tumours. Only triple-negative tumours were included, as almost all BRCA1-like^aCGH ^tumours are triple-negative. We compared this subset of 46 tumours for baseline clinicopathological variables, like T-stage, N-stage and grade, with all 149 TN samples of the randomized controlled trial. No difference in either variable was seen (data not shown).

**Figure 1:**
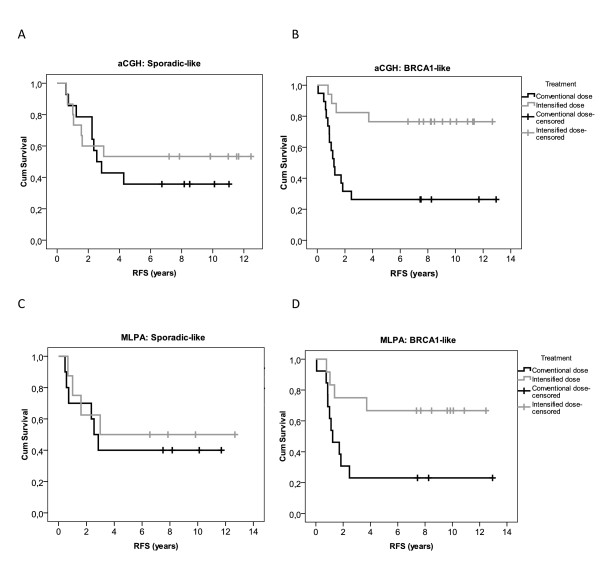
**Survival analysis**. Association of BRCA1-like classification with array Comparative Genomic Hybridisation (aCGH) and with Multiplex Ligation-dependent Probe Amplification (MLPA), with outcome after intensive alkylating chemotherapy and conventional dose chemotherapy. Kaplan-Meier survival curves according to the BRCA1 classification of tumours. The patients had been randomly assigned to either intensified dose chemotherapy or conventional dose chemotherapy. **(a) **Recurrence-free survival (RFS) of Sporadic-like CGH triple-negative (TN) patients (log rank test: *P *= 0.543), **(b) **RFS of BRCA1-likeCGH TN patients (*P *= 0.002), **(c) **RFS of Sporadic-like MLPA TN patients (*P *= 0.625), **(d) **RFS of BRCA1-like MLPA TN patients (*P *= 0.024).

Recurrence-free survival by MLPA analysis was analyzed, in the same way as previously reported for aCGH analysis (Figure 1) [10]. The same pattern was observed for the aCGH as for the MLPA classifier. The tumours with a Sporadic-like profile do not show a significant difference between conventional and intensive chemotherapy (Figure 1A, C). However, the BRCA1-like tumours show a significantly better recurrence-free survival after intensive chemotherapy than after conventional dose chemotherapy (Figure 1B, D) (*P *= 0.002 for aCGH and *P *= 0.024 for MLPA). Although not all triple-negative aCGH samples from the original publication [10] could be analyzed with MLPA due to limited material, the difference in recurrence free survival between BRCA1-like and Sporadic-like samples depending on whether or not intensive chemotherapy is given, remained (Figure 1).

## Discussion

In this study, we describe the design, testing and validation of a Multiplex Ligation-Dependent Probe Amplification (MLPA) kit to determine BRCA1-like genomic profiles. Our findings suggest that this classifier can be used both in clinical genetic testing and in treatment response prediction. In the clinical genetic setting, the classifier could be used in addition to conventional BRCA1 mutation testing: as a tool to classify BRCA1 variants of unknown significance or to identify potential BRCA1 mutations other than the mutations that are currently screened for. MLPA is a rapid, cost-efficient method, suitable for FFPE tissue derived DNA, and, therefore, potentially useful for routine clinical application.

In previous work, we developed an aCGH-based classifier recognizing the genomic pattern of BRCA1-mutated breast tumours. We showed that this classifier could identify hereditary breast tumours, for which no mutation was identified by routine clinical screening [6]. In addition, we showed that this BRCA1-like pattern was also observed in sporadic breast cancer patients, and was predictive for treatment benefit [10]. Currently, several prognostic tests for breast cancer are being evaluated in clinical studies or have already been FDA approved [22,23]. However, predictive tests seem to be more difficult to develop. Even more than for other breast cancer subtypes, there is an urgent need for targeted therapies for triple-negative tumours, as the only systemic treatment option for this tumour group continues to be chemotherapy. With the clinical introduction of the PARP inhibitors, hope has been raised that at least the triple-negative tumours defective in homologous recombination could benefit from this treatment [14,24]. Our MLPA test can recognize both BRCA1-mutated and sporadic tumours with a BRCA1-like genomic profile. We hypothesized that those tumours show BRCAness [13] and thus would be highly sensitive to this new class of targeted drugs. When we applied the MLPA assay to tumours of patients treated in a randomized controlled trial, we observed that patients with a BRCA1-like MLPA profile had a significantly better recurrence-free survival after intensified alkylating chemotherapy than patients with a Sporadic-like profile. Although the groups were small, this lends further support to the predictive power of our new MLPA BRCAness test, which appears to be as accurate as the original aCGH test.

The clinical application of a DNA test is associated with more requirements for practicability than an assay used for research purposes only. It must be rapid as the patient and the physician are in need of a result within days; it should be feasible using standard equipment; and it must be cost effective. In addition, it is an advantage if the test can be performed on FFPE tissue, as this is the material routinely available in clinical pathology laboratories. MLPA fulfils all these criteria. Therefore, we believe that this assay makes BRCAness testing a viable option for triple-negative tumours. The kit is now ready for prospective clinical validation and it can already be used along with aCGH in the clinical genetics testing mentioned previously.

Currently, there is no gold standard for BRCAness. In this study we used MLPA as a surrogate, in the previous, aCGH. Others have used gene expression [25], methylation [16] and RAD51 [26]. However, none of those methods is perfect and generally accepted for BRCAness determination, and in not all studies data were compared with confirmed BRCA1 mutation carriers. We have shown a high degree of concordance with the aCGH-based BRCA1-like classifier (94% in the training set and 86% in the test set). The concordance was, however, not perfect. In fact, the BRCA1-aCGH is not perfect as well as in the original publication two BRCA1-mutation carriers were not classified as BRCA1-like tumours [6]. Also, in subsequent series, some BRCA1 mutated cases were missed [10] and were not identified as BRCA1-like^aCGH^. When we compared misclassifications between the BRCA1 aCGH and MLPA classifiers we found that five BRCA1-mutated tumours were not classified as BRCA1-like with MLPA and five were missed by aCGH (not completely overlapping). Based on these numbers we can conclude that MLPA and aCGH perform approximately equally well in identifying BRCA1 mutation carriers. Interestingly, one of the misclassified BRCA1 mutated cases was positive for ER staining. About 5% of BRCA1-mutated tumours are ER+ [27]. We observed before (data not published) that ER+ mutation carriers show a different pattern of copy number changes than triple-negative mutation carriers. Also, the literature suggests that ER+ tumours in mutation carriers is a different entity of disease, as these tumours usually arise later in life and are less aggressive than the TN tumours [27].

Based on aCGH data, both a BRCA1- and a BRCA2-like genomic profile can be obtained [6,8]. A limitation of MLPA compared to aCGH is that information on a restricted number of genomic loci is obtained. A BRCA2-like profile cannot yet be obtained by MLPA for these tumours and 5 to 10% of triple-negative tumours show such a BRCA2-like profile and these may have the same hypersensitivity to intensive alkylating therapy (unpublished data). The translation of the BRCA2-profile to a MLPA kit will be a challenge, as it makes use of 771 probes, rather than the 191 probes of the BRCA1 classifier. MLPA is limited to a maximum of 50 probes, including reference probes, and ideally each important genomic region is covered by a minimum of two probes.

For our clinical workup we now use the following routine for triple-negative tumours. First, a MLPA for BRCA1-like pattern is performed. If the tumour shows a BRCA1-like^MLPA ^pattern, in the clinical diagnostics setting, we can continue with extra analysis sorting out the defect (that is, methylation analysis). In the therapy selection setting, the patient can be eligible for protocol treatment in an intensified alkylating chemotherapy study (for example, trial NCT01057069 [28]). This study also serves as a prospective validation of our MLPA assay. If the tumour shows a Sporadic-like profile, there is still the possibility that the tumour would be BRCA2-like at aCGH. Therefore, for these patients aCGH analysis could still be useful.

## Conclusions

In conclusion, we have shown that the BRCA1-like MLPA assay can be both applied in clinical genetic testing and in treatment benefit prediction. As the MLPA assay is rapid and robust, can easily be multiplexed, and works well with DNA derived from paraffin-embedded tissues, it is a suitable method for a clinical application. However, both its use in the clinical genetics as in the treatment prediction setting should be validated in large prospective series.

## Abbreviations

aCGH: array Comparative Genomic Hybridisation; DSB: double strand break; FFPE: formalin-fixed, paraffin-embedded; MLPA: Multiplex Ligation-dependent Probe Amplification; PAM: prediction analysis of microarrays; PARP: poly ADP ribose polymerase; TN: triple-negative.

## Competing interests

Nadja Laddach and Suvi Savola are employees of MRC-Holland. The other authors do not have competing interests.

## Authors' contributions

EHL, PMN and SR conceived and designed the study. EHL, NL, SPS, MAV AMMO, ALTI and JW acquired the data. EHL and LFAW analysed and interpreted the data. EHL, JW, PMN and SR prepared and revised the manuscript. All authors read and approved the final version of the manuscript.
